# Love Together, Parent Together (L2P2): a protocol for a feasibility study of a conflict reappraisal writing intervention for interparental couples with young children

**DOI:** 10.1186/s40814-022-01115-y

**Published:** 2022-08-06

**Authors:** Heather Prime, Amy Muise, Veronica Benyamin, Lehana Thabane, Mark Wade

**Affiliations:** 1 Department of Psychology, York University, 4700 Keele St, Toronto, ON M3J 1P3 Canada; 2 Health Research Methods, Evidence & Impact, McMaster University, 1280 Main St. W, Hamilton, ON L8S 4L8 Canada; 3 Biostatistics Unit, St Joseph’s Healthcare Hamilton, Hamilton, ON Canada; 4 Faculty of Health Sciences, University of Johannesburg, Johannesburg, South Africa; 5 Applied Psychology & Human Development, University of Toronto, 252 Bloor St. West, Toronto, ON M5S1V6 Canada

**Keywords:** Interparental conflict, Family systems, Writing intervention, Single-arm feasibility study

## Abstract

**Background:**

The COVID-19 pandemic has introduced or amplified stress and challenge within couples’ relationships. Among those who are particularly vulnerable to heightened conflict and lower relationship satisfaction during this time are interparental couples with young children, whose relationships may have already been tenuous prior to the pandemic. Stress within the interparental relationship may have ripple effects on all family subsystems and child adjustment. The Love Together Parent Together (L2P2) program is a brief, low-intensity writing intervention adapted for parents of young children that was designed to reduce conflict-related distress and prevent declines in relationship satisfaction. Based on an original writing intervention by Finkel and colleagues, L2P2 has adapted the intervention duration and study population to be appropriate to the current global context. This study will examine the key feasibility metrics related to this adapted program with the goal of identifying problems and informing parameters of future pilot and/or main RCTs.

**Methods:**

The current study is a non-randomized feasibility study, using a single-arm, pre-test/post-test design to primarily assess the feasibility of an evaluative RCT, and to secondarily assess the potential effects on outcomes to be used in a future RCT. Couples will be recruited through three community-based agencies with the goal of obtaining a socio-demographically diverse sample. The first 20 couples to enroll will be included. Baseline and post-intervention surveys will be conducted, and a writing intervention will take place (three 7-min sessions over the course of 5 weeks). The primary outcomes will be feasibility metrics of recruitment rates, appropriateness of eligibility criteria, sample diversity, retention, uptake, adherence, and acceptability. In addition, we will develop an objective measure of couple “we-ness” based on an analysis of writing samples. The secondary outcomes will include couples’ measures (i.e., relationship quality, perceived partner responsiveness, self-reported responsiveness, conflict-related distress), and additional family outcomes (i.e., parent-child relations, parental/child mental health). Criteria for success are outlined, and failure to meet the criteria will result in adaptations to the measurement schedule, intervention design, recruitment approach, and/or other elements of the program.

**Discussion:**

This feasibility study will inform several components of the procedures used for a subsequent pilot RCT, in which we will examine the feasibility of the methodology used to evaluate the program (e.g., randomization, attrition to follow-up assessment/across groups, and sample size estimation, preliminary effectiveness), as well as the main RCT, which will investigate the effectiveness of the intervention on primary outcome measures and mediating pathways.

**Trial registration:**

ClinicalTrials.gov, NCT05143437

**Supplementary Information:**

The online version contains supplementary material available at 10.1186/s40814-022-01115-y.

## Introduction

### Couples’ relationships during COVID-19: implications for young families

The COVID-19 pandemic has introduced heightened levels of stress for many couples that increase the risk of harmful dyadic processes such as decreased responsive support, hostility, and withdrawal [1]. Some couples’ relationships may be particularly vulnerable due to pre-pandemic stressors―namely, couples with young children. Indeed, the transition to parenthood is a challenging time, and negative changes to couples’ satisfaction often persist beyond the first year postpartum [2]. For instance, using a large and diverse community sample of mothers, one study found that more than 20% of mothers reported high and worsening relationship conflict over the early childhood period [3]. In the current pandemic, this developmental stage of parenthood is further threatened by distinct strains experienced by many parents. Pandemic-related stressors, including financial, family, and pandemic-specific factors, have been linked to parental mental health, particularly among mothers [4], with parents reporting higher levels of depression and anxiety [5] and more frequent use of alcohol as a coping strategy compared to non-parents [6]. Given the importance of individual mental health to the well-being of couples, this represents a time of acute stress to interparental relationships, which may have lasting effects.

Interparental relationships form the foundation of healthy family functioning, with strong evidence for spillover effects from the interparental relationship to other family systems [7, 8] and child adjustment [9]. This may be especially true under conditions of risk [10], including stress emanating from the pandemic [11]. As such, threats to the interparental relationship during this time represent a family-wide risk factor. It is therefore important to provide access to evidence-based interventions aimed to prevent the deterioration of interparental relationships during and after the pandemic, with implications for the entire family. The current protocol describes a study that will assess the feasibility and acceptability of an interparental intervention designed to combat declines in relationship quality amid this global crisis.

### Potential utility of a brief intervention

Prior to the pandemic, there were calls for increased translational research to inform large-scale couples’ interventions [12]. In the current context, the widespread and far-reaching threat of pandemic stress has made this need even more salient. Introducing novel, brief interventions to address couples’ relationships has the potential to mitigate accessibility challenges related to reach and retainment [13]. One brief intervention, deemed the “Marriage Hack,” targets maladaptive conflict patterns by encouraging couples to reappraise their disagreements from a neutral, third-party perspective [14]. This low-resource intervention, which involves three 7-min writing sessions (for a total of a 21-min intervention), has been shown to buffer against normative declines in marital quality over time by reducing conflict-related distress. Such an intervention has the potential for widespread scale-up among couples at risk for relationship deterioration. In addition to its brief nature, it also has the advantage of being a fully online intervention, and will therefore reduce access barriers inherent to the pandemic and to couples with young children.

### The need for a feasibility study

The overarching goal of this research program is to evaluate the effectiveness of the Marriage Hack intervention using a randomized controlled trial (RCT) with a new sample―couples of young children. In addition, we will examine the impact of the intervention on the interparental relationship as well as other family relationships, which was not a goal of the original intervention.

The current intervention resembles the original intervention in almost all aspects, with the exception of the timing of the intervention. Specifically, to optimize reach and retainment, we will adapt the intervention from its original 12-month course to run over a 5-week timeframe. The number of writing sessions will remain the same (i.e., three sessions) but will be expedited to one session every 2 weeks. To this end, there is strong evidence that brief and precise relationship interventions that aim to alter specific psychological processes―such as conflict reappraisal―can lead to significant benefits over time, including in higher-risk samples [15].

The original study was conducted with a relatively low-risk sample in terms of risk for couple-related distress. The current study is considered a secondary prevention program in that we are targeting couples at risk for relationship difficulties based on the developmental stage (couples with young children) and context (a global crisis). We expect this intervention to be effective for this new study population, as poor cognitive reappraisal skills are an important contributor to marital dissatisfaction in high-risk couples [16], and they buffer against the negative impact of marital conflict on marital satisfaction in a range of couples (i.e., newly married and remarried) [17]. Taken together, the use of a *brief* conflict reappraisal intervention to address negative conflict dynamics is expected to lead to benefits for couples in distress, including parents with young children.

Best practice for establishing effectiveness through an RCT requires a step-wise approach: (1) a feasibility study to address specific elements of the RCT (e.g., intervention characteristics), (2) a pilot RCT to address barriers and inform parameters of the main RCT, and (3) the main RCT to assess effectiveness [18]. The current protocol describes a non-randomized, single-arm feasibility study (step 1 above), with the aim to assess feasibility, identify and rectify problems, and increase the success of a future evaluative RCT.

### Objectives

The primary aim of the current study is to assess the feasibility and other methodological components of the Love Together, Parent Together (L2P2) intervention to inform the parameters of a future pilot (step 2 above) and main RCT (step 3 above). L2P2 is a brief conflict reappraisal program for couples with young children designed to support whole family functioning. The primary objectives of the current feasibility study are as follows:

1. *Recruitment:* Establish partnership with three recruitment sources and examine recruitment rates to determine if additional recruitment sources are needed.
2. *Sample:* Assess whether our recruitment approach and eligibility criteria are appropriate for the intended sample (i.e., participants with mild to moderate levels of couple distress as in a secondary preventive intervention). Relatedly, we are interested in the heterogeneity of the sample obtained through this recruitment approach based on sample demographics including income/education level, racial/ethnic identification, immigration status, and sexual orientation/gender identity.
3. *Program:* Assess program retention, adherence, and uptake rates―that is, the extent to which participants complete the assessment schedule in full, engage in the three writing (intervention) sessions, and report the use of conflict-reappraisal strategies in between sessions.
4. *Measurement:* Conduct a preliminary validation of a primary outcome measure of couple “we-ness” [19] based on a content analysis of writing samples collected during the intervention. This new measure will be used in conjunction with pre-existing self-report measures of perceived partner responsiveness and responsiveness towards one’s partner for a multi-method/multi-informant assessment approach in subsequent pilot/main RCTs.
5. *Acceptability*: Examine the acceptability of the adapted intervention. We will examine whether the acceptability of the intervention varies as a function of key sociodemographic variables such as gender, race/ethnicity, and immigration status.

The secondary objective is to explore preliminary effects on couples’ measures (i.e., relationship quality, perceived partner responsiveness, self-reported responsiveness, conflict-related distress), and other family outcomes (i.e., parent-child relations, parent/child mental health) to see if the expected changes are evident following participation in the intervention.

## Methods

The current protocol is written in accordance with *a guide to the reporting of protocols of pilot and feasibility trials* [20], *guidelines for reporting non-randomized pilot and feasibility studies* [21], and the *CONSORT extension to pilot and feasibility trials* [22], with adaptations for the current non-randomized design. In addition, we have adhered to the SPIRIT guidelines for reporting protocols. The current protocol was registered on ClinicalTrials.gov (NCT05143437), where amendments to the protocol will also be documented.

### Study design

The current study is a non-randomized feasibility study, using a single-arm, pre-test/post-test design. Couples will be recruited via three recruitment platforms via email listservs. The first 20 couples who meet the eligibility criteria and consent to the research process (self-directed, online) will be included in the study. Baseline assessments will include surveys collecting information on participant sociodemographic characteristics, COVID-19-related stress, self-report measures of couples’ distress, relationship quality, parenting practices, responsiveness directed towards partner, and perceived partner responsiveness, as well as the mental health of self and one target child. The intervention will take place over 5 weeks, with a total of three 7-min writing intervention sessions. At the beginning of each intervention session, participants will complete a brief survey of conflict frequency, as well as the use of conflict reappraisal strategies since the last session. A post-intervention survey will include all measures from baseline (except COVID-19-related stress and demographics), in addition to an acceptability survey.

### Participants

Eligibility criteria are as follows: (i) both participants endorse being in a relationship, (ii) partners reside in the same home, (ii) one or more children under the age of 6 living at home, (iii) both participants are over age 18 years, and (iv) both members of a couple agree to participate. Exclusion criteria included (i) no current plans or history of separation or divorce (as this is meant to be a secondary preventative intervention for couples experiencing mild-to-moderate but not severe relationship difficulties). Of note, the participant pool was considered at-risk due to developmental stage (couples with young children) and context (pandemic); however, we did not screen for relationship distress for the purposes of eligibility criteria. Instead, we plan to look at this descriptively (i.e., proportion of couples with mild-moderate risk for relationship distress).

Recruitment sources, who were involved from the outset of the study and consulted on study design, include (1) Moms at Work, a community, education, and advocacy group supporting women in their careers; (2) Unemployed Help Centre of Windsor Inc., a nonprofit organization assisting the un/underemployed and disadvantaged person during the transition period in the reemployment process; and (3) EarlyOn Child and Family Centres, who offer free, high-quality programs for young families. These recruitment sources were selected to get a range of participants in terms of potential risk factors for relationship problems.

Listserv members from each recruitment source will receive an invitation to participate in research with a direct contact link to the L2P2 study platform via Qualtrics. Participants will go through an eligibility screen, followed by a review of a letter of information and informed consent. Once participants have consented, they will be asked for their contact information and that of their partner (if they are the first member of the couple to sign up). The second member of the couple will be contacted directly by the study team via email with an invitation to enroll. Once both members of a couple have enrolled, they will be given couple and participant IDs, and a survey schedule will be set up and executed. For a schematic diagram of the time schedule of enrollment, intervention, and assessments, see Table 1.

**Table 1:** Schedule of enrollment, intervention, and assessments

	Study period					
	Enrollment	Post-enrollment				
Time point	*−t*_*1*_	*t*_*1*_ *Week 0*	*t*_*2*_ *Week 1*	*t*_*3*_ *Week 3*	*t*_*4*_ *Week 5*	*t*_*5*_ *Week 6*
Enrollment						
Eligibility screen	X					
Informed consent	X					
*Contact information*	X					
Allocation						
Interventions						
*Love Together Parent Together intervention*			X	X	X	
Assessments						
*Demographics, COVID-19 stress, relationship distress*		X				
*Couples’ relationship quality, perceived partner responsiveness, responsiveness to partner, parent mental health, child mental health, parent-child relations*		X				X
*Conflict-related negativity*			X	X	X	
*Use of conflict reappraisal strategy*				X	X	
*Acceptability*						X

### Intervention

Given that this is a single-arm design, only an intervention group will be described. The intervention sessions will consist of two stages. First, participants will be asked to provide a “summary of a time when you and your romantic partner did not agree in the last 2 weeks...” focusing on “you and your partner’s actions (what you said and did), not about what you were thinking or feeling.” Second, participants will be asked to engage in a writing task wherein they will be instructed to reappraise the conflict they previously reported based on the following prompts [14]:

“Think about this disagreement with your partner from the perspective of a third party who wants the best for all involved. This is a person who sees things from a neutral point of view. How might this person think about the disagreement? How might this person find the good that could come from it?”

“Some people find it helpful to take this third-party perspective when they are with their romantic partner. However, almost everybody finds it hard to take this third-party perspective at all times. In your relationship with your partner, what might make it hard to take this third-party perspective, especially when you’re having a disagreement with your partner?”

“Even though it is hard to take a third-party perspective, people can do it. Over the next two weeks, please try your best to take this third-party perspective when you are with your romantic partner, especially during disagreements. How might you be able to take this perspective in your interactions with your partner over the next two weeks? How might taking this perspective help you make the best of disagreements in your relationship?”

Participants will receive email reminders 1 week after each intervention session to prompt the use of the reappraisal task. Email reminders first remind them of the prompts they wrote about during the writing session. Participants are then encouraged to use the conflict reappraisal strategy, as follows:

“As you go through your daily life, please keep in mind the benefits of adopting a third-party perspective in your romantic relationship. Sometime today (now, if the timing works), please take a few moments to think about ways you can take this point of view about conflicts in your romantic relationship.”

There are no criteria for discontinuing or modifying allocated interventions. Participants will be informed that they can withdraw from participation in the study at any time and that they do not need to complete all questions in surveys or writing sessions. Participants who withdraw prematurely will be compared to completers on baseline demographic and relationship characteristics.

Several steps will be taken to enhance recruitment efforts and applicability of the intervention to the study population and to improve adherence to intervention protocols. These steps were developed, in part, through consultation with recruitment partners (listed above). Recruitment flyers will be distributed online and in person. The study will be conducted online to reduce barriers to participation. The survey and intervention sessions will be made available for mobile and/or computer users to enhance access and promote adherence. The language used in study materials (e.g., writing prompts) will be simplified (i.e., the use of terms such as “disagree” rather than “conflict”). Remuneration of participants will occur as the study progresses (i.e., after each session) via email in the form of a choice of gift card. Specifically, each participant will receive $5.00 for the baseline survey, $10.00 for each of the three intervention sessions, and $5.00 for the post-intervention survey ($40.00 per participant for full participation). Reminders of study timelines and survey/intervention session expiry dates will be provided by email.

### Outcomes and analysis

Table 2 presents all the study objectives with associated outcomes and criteria for success of feasibility, hypothesis for secondary outcomes, and methods of analyses, when applicable. Feasibility outcomes will be reported descriptively using descriptive statistics, means (standard deviations), and frequencies/percentages. Analyses for pre-post change and validation of an objective primary outcome measure are described in Table 2. If success indicators are not met, we will make changes to the study design accordingly (e.g., expand recruitment sources, adjust eligibility criteria, adapt intervention schedule, and/or compensation). Analyses will include all participants who complete baseline assessments. We will use Bayesian estimation, which is more robust with small sample sizes [32].

**Table 2:** Study objectives with associated outcomes and criteria for success of feasibility, hypothesis for secondary outcomes, and methods of analyses

	Outcome	Criteria for “success” of feasibility/hypothesis	Method of analysis
Primary objectives to determine:			
Recruitment	Number of participants *accessed* (i.e., initiate registration) per week, stratified by recruitment source	10 couples per week over the course of 4 weeks who access our registration site.	Descriptive statistics
	Number of participants *enrolled* per week, stratified by recruitment source	5 couples per week over the course of 4 weeks who enroll in the study.	
Eligibility criteria	% interested participants that meet the *inclusion criteria* (with reasons for exclusion)	< 50% of participants are excluded for any one criterion.	
Sample diversity	% participants income ≤ regional median, ≤ high school degree	> 30% of our sample has 1+ indicator.	
Sample diversity (race/ethnicity/immigration)	% participants racialized, immigrant	> 30% of our sample has 1+ indicator.	
Sample diversity (sexual orientation/gender)	% participants non-heterosexual, gender non-conforming	> 30% of our sample has 1+ indicator.	
Mild-moderate risk for relationship distress	% participants scoring ‘clinically distressed’ (<12) on the Brief Dyadic Adjustment Scale [23] % participants scoring “high” (> 29) on the *COVID-19 Family Stressor Scale* [24]	< 50% of eligible participants.	
Participant retention	% participants who remain in study until end of post-intervention assessment	> 90% of participants.	
Participant adherence	% participants who complete 2/3 intervention sessions	> 90% of participants.	
Participant uptake	% participants reporting some use of conflict reappraisal outside of sessions	> 80% of participants.	
Acceptability	% of participants reporting at least “good” on 80% or more indicators on an *Implementation Acceptability Scale* assessing attitude, burden, perceived effectiveness, and ethicality [25]	> 80% of participants, (stratified by gender, immigrant status, and racialized groups).	
Primary outcome measure development	Objective assessment of we-ness [19] based on content analysis of writing samples. Will include an analysis of first-person plural pronouns (we, us, our, ours), reference to partners’ internal states (beliefs, desires, intentions), and positive affective language [26]	Inter-rater reliability (Cronbach’s alpha > .80). Internal consistency (Cronbach’s alpha > .70). Significant group differences between male and female participants based on *t*-tests (*p* < .05). Significant correlations (*p* < .05) with indices of convergent validity (i.e., self-reported responsiveness, partner reported perceived partner responsiveness).	
Secondary objective to explore:			
Pre-post change in outcome measures	*Couples’ relationship quality:* Perceived Relationship Quality Components(PRQC) Inventory [27].	Intervention will improve outcomes from baseline to post-intervention surveys.	3-level multilevel models, similar to regression analysis but accounting for clustering within the data structure
	*Conflict-related negativity*: two items following fact-based summary: “I was angry at my partner for his/her behavior during this conflict,” “My partner’s behavior during this conflict was highly upsetting to me” [14].		
	*Perceived partner responsiveness/responsiveness directed towards partner*: two eight-item scales will assess participants’ perceptions of their partners’ responsiveness/insensitivity and their own responsiveness/insensitivity towards their partner, respectively [28].		
	*Parent-child relations*, Parenting Practices Scale from the Ontario Child Health Study [29].		
	*Parent Mental Health*, K10 Psychological Distress Scale [30].		
	*Child emotional and behavioral problems*, Pediatric Symptom Checklist (baby, preschool, and standard versions) [31].		

### Sample size

The targeted sample size for the current study is 20 couples. This was considered sufficient to identify a pattern in rates of recruitment over several weeks (expected five couples per week) and to offer a large enough sample to gauge the appropriateness of eligibility criteria and reasons for exclusion, diversity of sample, retention, adherence, and uptake. In addition, 20 couples are sufficient for accurate Bayesian estimation in the context of multilevel modeling [32], which will be used for preliminary analyses examining pre-post change in future study outcomes. We kept the sample size to a minimum for the purposes of reserving study resources for a planned pilot RCT.

The current protocol does not include stopping or discontinuing guidelines.

### Data management

No data entry is required as participants provide responses directly into Qualtrics. Study protocols have been developed by the principal investigator (PI: first author) which outline clear steps for enrolling study participants and scheduling surveys and compensation schedules. Data quality checks will be conducted at regular check points throughout data collection by the PI. Steps will be taken to ensure linking of participants within couples and over time and independent completion of surveys. Embedded data will be used across surveys to ensure consistency in reporting on specific children. Range checks will be conducted on all data to ensure valid values.

### Ethics and dissemination

Ethics approval has been granted by York University (Certificate #: e2021-266). Changes to the protocol will be reported in the publication of the study findings. Informed consent will be completed online (see Additional file 1).

All information provided by participants will be kept private and confidential. An electronic file linking participant contact information with their couple identification numbers will be only accessible by the PI (first author) and research coordinator. Qualtrics servers will contain email addresses linked to each participant’s data to allow for communication with participants (i.e., sending out reminders and surveys). Any data shared with the research team will contain only the participant codes and will be securely transferred using an encrypted email or secure server.

All study data will be temporarily stored on Qualtrics before anonymized records are sent to York University secure servers (OneDrive). Qualtrics is protected by high-end firewall systems. Qualtrics uses Transport Layer Security (TLS) encryption (also known as HTTPS) for all transmitted data. Anonymized data will be made available to the senior investigators (first, second, and senior authors) and their research assistants and students (current and future) under the direct supervision of the research team.

We will destroy any personally identifiable data at the end of the study. We will keep non-identifiable data to comply with open science and data sharing practices, as well as to allow for future analysis of data.

Dissemination plans include traditional outlets (e.g., peer-review journals and conferences), and sharing with the general public through social media and community talks (in conjunction with recruitment partners). American Psychological Association “Publication Practices and Responsible Authorship” guidelines will be used to determine authorship.

## Discussion

The main objective of the current feasibility study is to examine the feasibility, acceptability, and practicality of an adapted intervention designed for couples with young children during and after the COVID-19 pandemic. Considerations relate to processes, resources, and scientific factors such as recruitment rates, appropriateness of eligibility criteria and sample diversity, retention, adherence, and uptake, primary outcome measure development, and preliminary examination of change in outcome measures. We will also conduct a participant survey to assess the acceptability of the intervention program, stratified by subgroups.

Findings will inform the parameters and research protocol of a future pilot RCT, the aim of which will be to assess the feasibility of examining the effectiveness of the intervention program in a subsequent main RCT. Specifically, we expect the results of this feasibility study to inform what changes may need to occur, if any, prior to executing the pilot RCT.

In the subsequent pilot and main RCTs, we will be able to ask more nuanced questions about whether “wise interventions”―that is, brief and precise interventions that target specific psychological processes―can be used with higher-risk samples to address coercive relationship dynamics, with potential benefits over time. To this end, our broader research program will address questions about whether there is differential effectiveness of such interventions on couples based on initial risk levels (e.g., pandemic-related stress and/or dyadic adjustment), which will speak to the utility of using the intervention with low, mild-moderate, and high-risk couples. The program of research will also answer questions about the potential cascading effects of wise interventions not only within individuals (intra-individual effects), but between partners of a couple (inter-individual effects), and extending to other family systems (e.g., coparenting and parent-child relationships). Finally, findings from the broader research program will inform the rapidly evolving literature on technology-assisted family-based interventions. For instance, there is some evidence that technology-assisted parenting interventions are optimized when individuals have direct contact with a practitioner [33]. The current research program will address whether an online, self-directed couples’ relationship intervention is sufficient for enhancing couples’ relationship quality, with findings informing future iterations of the program, including the potential benefits of direct contact with a facilitator.

With appropriate adaptations, the Marriage Hack presented by Finkel and colleagues (2013) has the potential to meet a pressing need for a widescale program to prevent the sequelae of the COVID-19 pandemic on interparental relationships, family functioning, and child adjustment. The current feasibility study is a critical first step to ensuring the successful implementation of future pilot and main RCTs that will investigate the effectiveness of using this intervention with a new population (couples with young children) and in the context of an ongoing pandemic and its aftermath.

## Supplementary Information

**Additional file 1.** Informed consent.

## Data Availability

Not applicable
