# Supplementary material for: Love Together, Parent Together (L2P2): a protocol for a feasibility study of a conflict reappraisal writing intervention for interparental couples with young children
Source: Pilot Feasibility Stud. 2022 Aug 6;8:170. doi: 10.1186/s40814-022-01115-y (PMC9356452; doi:10.1186/s40814-022-01115-y)
Supplement: Supplementary file 1 — Additional file 1. Informed consent. [file 40814_2022_1115_MOESM1_ESM.pdf]

Letter of Information

Study Name:
Love Together, Parent Together (L2P2): Establishing a Brief Intervention for Couples with Young Children

Principal Investigator:
Dr. Heather Prime Assistant Professor, Department of Psychology, York University; mailing address: Behavioral Sciences Building, 4700 Keele Street Toronto, ON M3J 1P3;
email: hprime@yorku.ca

Co-Principal Investigators:
Dr. Mark Wade Assistant Professor, Department of Applied Psychology and Human Development, University of Toronto, m.wade@utoronto.ca

Dr. Amy Muise Assistant Professor, Department of Psychology, York University, muiseamy@yorku.ca

Purpose of the Research: This study will assess the feasibility of using a writing program with couples who have young children during and after the COVID-19 pandemic. The couples' writing program has been previously used to help relationship quality. In this study, we are adapting the program and examining how practical it is for couples and how they like it. We will study the processes we use to conduct the research, such as asking participants to fill out multiple surveys, to help inform future studies on this writing program. In the future, we plan to study if the adapted program helps couples' relationships and the family.

To be eligible for the study, you must:

1. Be 18+ years old
2. Be living with your romantic partner
3. Be in a romantic relationship with your partner
4. Have one or more children under 6 years old residing in the home
5. NOT have a history of or current plans to separation/divorce with your current partner

What You Will Be Asked to Do in the Research:
Part 1: After reading this form and providing informed consent to participate, you and your partner will each be asked to complete baseline surveys which will ask you about your demographic information, stressors related to the COVID-19 pandemic, your couple relationship, parenting practices, and the mental health of yourself and one of your children. This survey should take approximately 14 minutes to complete.

Part 2: You will engage in an intervention over a four-week period with three brief writing tasks (once every two weeks). These writing tasks are designed to teach you a strategy to help you think about relationship conflict from a neutral, third-party perspective. At the beginning of each session, you will complete a brief survey, and then you will engage in the brief writing task. In

total, each intervention session will take approximately 15 minutes to complete. In between sessions, we will send you an email with a reminder to use the strategy.

Part 3: After the four-week intervention, you will complete a post-intervention survey asking you about your couple relationship, parenting practices, and the mental health of yourself and your children. We will also ask you questions about your experience of the writing program and the study. This survey should take approximately 10 minutes to complete.

Compensation: Each partner will have the opportunity to receive up to $40 (CAD) in gift cards from Amazon, Starbucks, or Best Buy for completing the study (sent by email). Specifically, you will receive $5 for the baseline survey (Part 1; compensation sent during week 0), $10 for each intervention session for a total of $30 (Part 2; compensation sent during weeks 1, 3, and 5), and $5 for the post-intervention survey (Part 3; compensation sent during week 6). Participants will be compensated for any portion of the surveys they start or complete.

Risks and Discomforts: It is not uncommon for couples to experience distress and family challenges, particularly during times of stress (such as the COVID-19 pandemic). There is a small, short-term risk that you may experience discomfort or distress due to the personal nature of some of the questions included in the questionnaires (e.g., those related to relationships, parent and child mental health, and parenting practices). Additionally, participation in the writing program may bring greater awareness to your couples' conflict dynamics. The likelihood of these risks is low. You may skip any question and withdraw from the study at any time without penalty.

Benefits of the Research and Benefits to You: You may enjoy participating in this type of study as it is an opportunity to reflect on your family dynamics. You may also benefit by learning more about the research process. Additionally, the writing program may improve your conflict resolution skills. It is possible, however, that participation in this study will be of no direct benefit to you. Your participation will contribute to the research on supporting relationship functioning and family well-being during and after COVID-19.

Voluntary Participation and Withdrawal: Participation in this study is completely voluntary and you may choose to stop participating at any time. Your decision not to volunteer, to stop participating, or to refuse to answer questions will not influence the nature of the ongoing relationship you may have with the research team and York University either now, or in the future. If you stop participating, you will still be eligible to receive compensation for started or completed procedures. Should you wish to withdraw after the study, you will have the option to also withdraw your data up until the end of the study (at which point removing your data will not be possible). If you choose to discontinue study participation at any point, please contact the study email primefamilylab@yorku.ca. Also, opt out links can be found in all emails received.

Confidentiality: Confidentiality will be provided to the fullest extent possible by law. All data will be coded by participant number rather than by name. Your name/personal information will not appear in any report or publication of the research. All study data will be temporarily stored on Qualtrics (an online survey platform protected by high-end firewall systems and encryption for all transmitted data) before being sent to a secure server at York University. In the situation

that data must be downloaded directly to a device, the device will be password protected. Only research staff/research team members, which includes the investigators listed at the top of this form and their students/research assistants (present and future), will have access to this information. We will destroy any personally identifiable data (participant names, telephone numbers, and email addresses) at the end of the study. We will keep non-identifiable data to allow for future analysis of data.

Future Research: The data collected in this research project may be used – in an anonymized form - by members of the research team in subsequent research investigations exploring similar lines of inquiry. Such projects will still undergo ethics review by the HPRC, our institutional Research Ethics Board. Any secondary use of anonymized data by the research team will be treated with the same degree of confidentiality and anonymity as in the original research project.

Online Surveys: The researcher(s) acknowledge that the host of the online survey (Qualtrics) may automatically collect participant data without your knowledge (i.e., IP addresses). Although this information may be provided or made accessible to the researchers, it will not be used or saved on the researchers' system without your consent. Further, because this project employs e-based collection techniques, data may be subject to access by third parties as a result of various security legislation now in place in many countries and thus the confidentiality and privacy of data cannot be guaranteed during web-based transmission.

Questions About the Research? If you have questions about the research in general or about your role in the study, please contact the study team (primefamilylab@yorku.ca). This research has received ethics review and approval by the Human Participants Review Sub-Committee, York University's Ethics Review Board and conforms to the standards of the Canadian Tri-Council Research Ethics guidelines. If you have any questions about this process, or about your rights as a participant in the study, please contact the Sr. Manager & Policy Advisor for the Office of Research Ethics, 5th Floor, Kaneff Tower, York University (telephone 416-736-5914 or e-mail ore@yorku.ca).

Couples Resources: It is possible that thinking about your romantic relationship may bring up some uncomfortable memories, thoughts, or emotions. These feelings are completely normal. If you'd like to talk to someone about any issues that come up during the study, you may consider contacting a mental health professional. You can find a psychologist through your province's Psychological Association (http://www.cpa.ca/public/whatisapsychologist/PTassociations/)

Additional Support Resources: If you or a family member experience a crisis or require mental health support, we encourage you to access one of these resources.

Support for adults experiencing a mental health crisis
Crisis Services Canada: 1-833-456-4566

Counselling support for First Nations and Inuit.
Hope for Wellness Help Line: 1-855-242-3310

Resources for mental health and social support

Ementalhealth.ca

Counselling, information and referrals, and text-based support to young people.
Kidshelpphone.ca

We encourage you to save or print a copy of this form for your records.

Consent
Providing consent means you have understood the nature of this project and wish to participate. It also means you understand that you can stop participation at any time without consequence. I have read the letter of information and consent to participate in this study. I have read the letter of information and do not consent to participate in this study.

- ☐ I have read the letter of information and consent to participate in this study.
- ☐ I have read the letter of information and do not consent to participate in this study.
